# On the Baliga’s Figure-Of-Merits (BFOM) Enhancement of a Novel GaN Nano-Pillar Vertical Field Effect Transistor (FET) with 2DEG Channel and Patterned Substrate

**DOI:** 10.1186/s11671-019-2960-8

**Published:** 2019-04-11

**Authors:** Zeheng Wang, Zirui Wang, Zhenwei Zhang, Di Yang, Yuanzhe Yao

**Affiliations:** 1 School of Information and Software Engineering, University of Electronic Science and Technology of China, Chengdu, 610054 People’s Republic of China; 2 School of Electronic Science and Engineering, University of Electronic Science and Technology of China, Chengdu, 610054 People’s Republic of China

**Keywords:** GaN, FET, Nano-pillar, Patterned substrate

## Abstract

A novel enhancement-mode vertical GaN field effect transistor (FET) with 2DEG for reducing the on-state resistance (*R*_ON_) and substrate pattern (SP) for enhancing the breakdown voltage (BV) is proposed in this work. By deliberately designing the width and height of the SP, the high concentrated electric field (E-field) under p-GaN cap could be separated without dramatically impacting the *R*_ON_, turning out an enhanced Baliga’s Figure-Of-Merits (BFOM, BV^2^/*R*_ON_). Verified by experimentally calibrated ATLAS simulation, the proposed device with a 700-nm-long and 4.6-μm-width SP features six times higher BFOM in comparison to the FET without patterned substrate. Furthermore, the proposed pillar device and the SP inside just occupy a nano-scale area, enabling a high-density integration of such devices, which renders its high potential in future power applications.

## Background

Nowadays, wide bandgap semiconductors such as ZnO, In_2_O_3_, SiC, and gallium nitride (GaN) have attracted attention [1–5]. Whereas, considering the electronic properties, the lateral AlGaN/GaN high electron mobility transistor (HEMT) is widely considered as a potential candidate for substituting the Si-based device in power or frequency applications due to the higher breakdown voltage (BV) as well as the stronger thermal stability. A lot of efforts, such as p-type cap [6, 7], fluorine ion implantation [8, 9], thin barrier [10, 11], double channel [5, 12], and field-coupled gate [13], have been made on the realization of the enhancement-type HEMT that is desired to simplify the driver circuit.

These technologies face, however, many formidable challenges such as low uniformity of the threshold voltage, the waste of vertical chip area, current collapse, limited Baliga’s Figure-Of-Merits (BFOM), and so on. Especially, the contradiction between the drift length and the BV negatively influences the scaling-down of the device [14, 15]. In other words, smaller device leads to lower BV, in which it is harder to adopt the junction terminals that promote the BFOM by optimizing the electric field distribution. To this end, back barrier [16], buried junction [17], quantum well field plate [18], and other structures that are inserted into the lateral HEMT exhibiting the feature of the electrical field plate have been proposed to enhance BV by utilizing the vertical region of the chip.

On the other hand, by the virtue of the superior natures of GaN, the bulk GaN vertical field effect transistor (VFET) attracts more and more attention due to the easier realization of enhancement-type functionality and the full utilization of the vertical region [19–22]. Many novel structures are presented by experiments or simulations to incline the BV and simultaneously reduce the on-state resistance (*R*_ON_) [23–25]. However, not to mention the difficulties in fabricating the super-junction (SJ) in GaN, the lack of the high-mobility two-dimensional electron gas (2DEG) leads to a higher *R*_ON_ [26], which hinders the optimization of BFOM in such devices.

In this work, a novel enhancement-mode vertical GaN FET with 2DEG for reducing the *R*_ON_ and substrate pattern (SP) for enhancing the BV is proposed, wherein the combination of the 2DEG channel and the SP effectively balances the contradiction between the low on-state resistance and the high BV. Furthermore, the proposed device pillar and the SP inside just occupy a nano-scale area, enabling a high-density integration of such devices. Verified by numerical simulation constructed in ATLAS, the proposed device features higher BFOM compared with the same field effect transistor (FET) without the patterned substrate, rendering its high potential in future power applications.

## Method

**Fig. 1:**
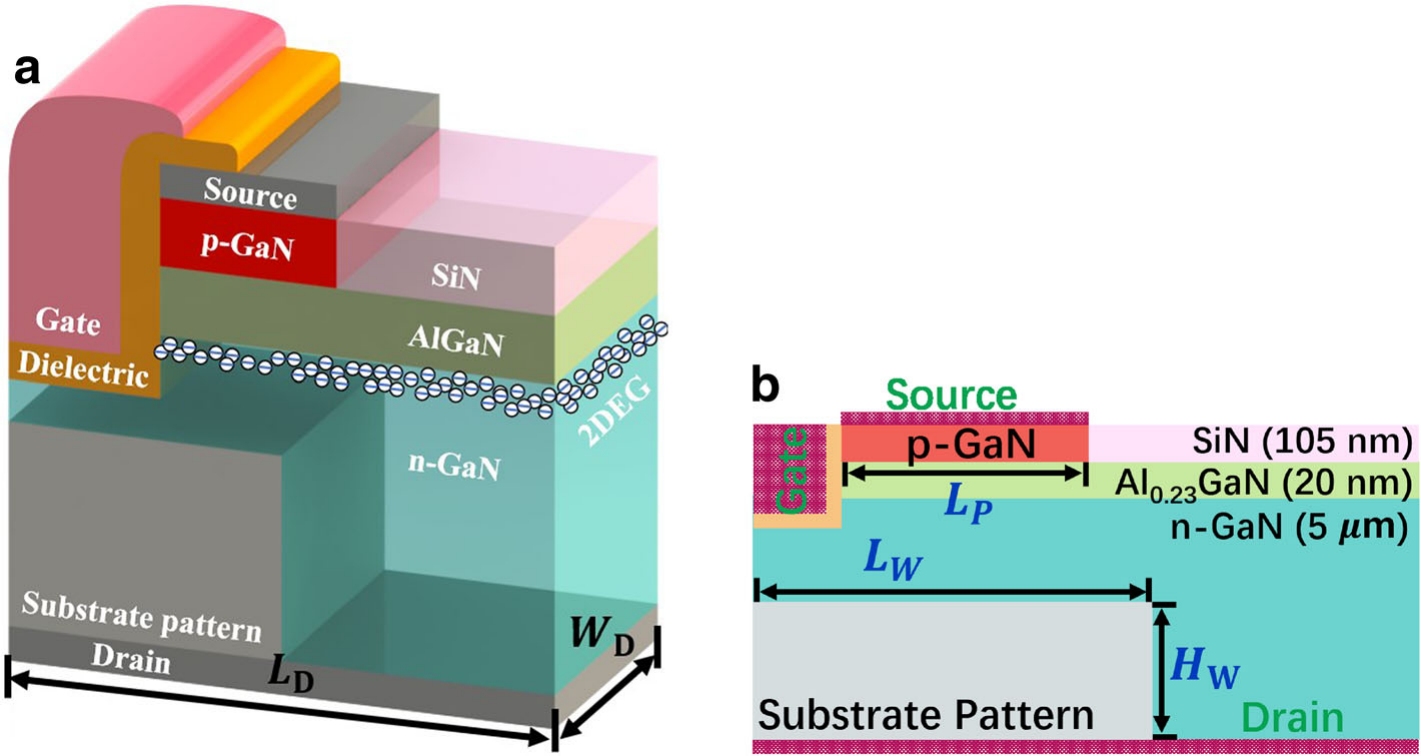
The schematic **a** 3D pillar structure and **b** cross-section with labeled geometric parameters of the proposed SP-VFET

The proposed device is generated in a normal Al_0.23_GaN/GaN wafer with a highly concentrated n-type substrate acting as the drain electrode as shown in Fig. 1a, where the thickness of the layer silicon nitride (SiN), AlGaN, and GaN are 105 nm, 20 nm, and 5 μm, respectively. A n-type GaN with 2 × 10^16^ cm^−3^ doping *n*_D_ and a p-type GaN cap with 2 × 10^17^ cm^−3^ doping *n*_A_ is set as the buffer and the composite channel respectively [27, 28]. Another component of the channel beside the gate is a thin AlGaN layer which is introduced for inducing 2DEG as shown in Fig. 1b. A SP, made by aluminum oxide (Al_2_O_3_) for example in this paper, is grown on the substrate.

The whole device therefore could be fabricated by a standard process successively: (1) the epitaxial deposition of the conduction substrate and the integrate SP layer, (2) the partial etching of the SP pattern, (3) the deposition and polishing of n-GaN buffer, (4) the deposition of AlGaN barrier and p-GaN cap, and (5) the fabrication of electrodes and passivation.

The implanted ATLAS simulator is calibrated by the experimental data from an enhancement-type HEMT with a p-GaN cap [29, 30]. The calibrated and other specifications of the device are shown in Table 1. Other configurations could be found in our previous work [31]. Type and density of the interface trap located at the SP/GaN interface are referred to capacitance-based experimental measurements [32–34]. The polarization charge on the AlGaN/GaN surface is confirmed according to the corresponding simple quadratic fitting equation [35].

**Table 1:** Device specifications

Parameter	Value and unit
Device length	*L*_D_ = 1 μm
Device depth	*W*_D_ *=* 1 μm
Polarization charge	σ_p_= 6.5 × 10^12^ cm^−2^
SP interface trap (Al_2_O_3_)	*D*_SP_ *=* 8 × 10^12^ cm^−2^; *E*_T_ *= E*_C_-0.5 eV
p-GaN cap length	*L*_P_ = 0.4 to 0.7 μm
SP length	*L*_W_ = 0 to 800 nm
SP height	*H*_W_ = 0 to 4.7 μm
Gate length	*L*_*G*_ *=* 15 nm
Gate height	*L*_W_ = 0.17 μm

## Physics Mechanism

**Fig. 2:**
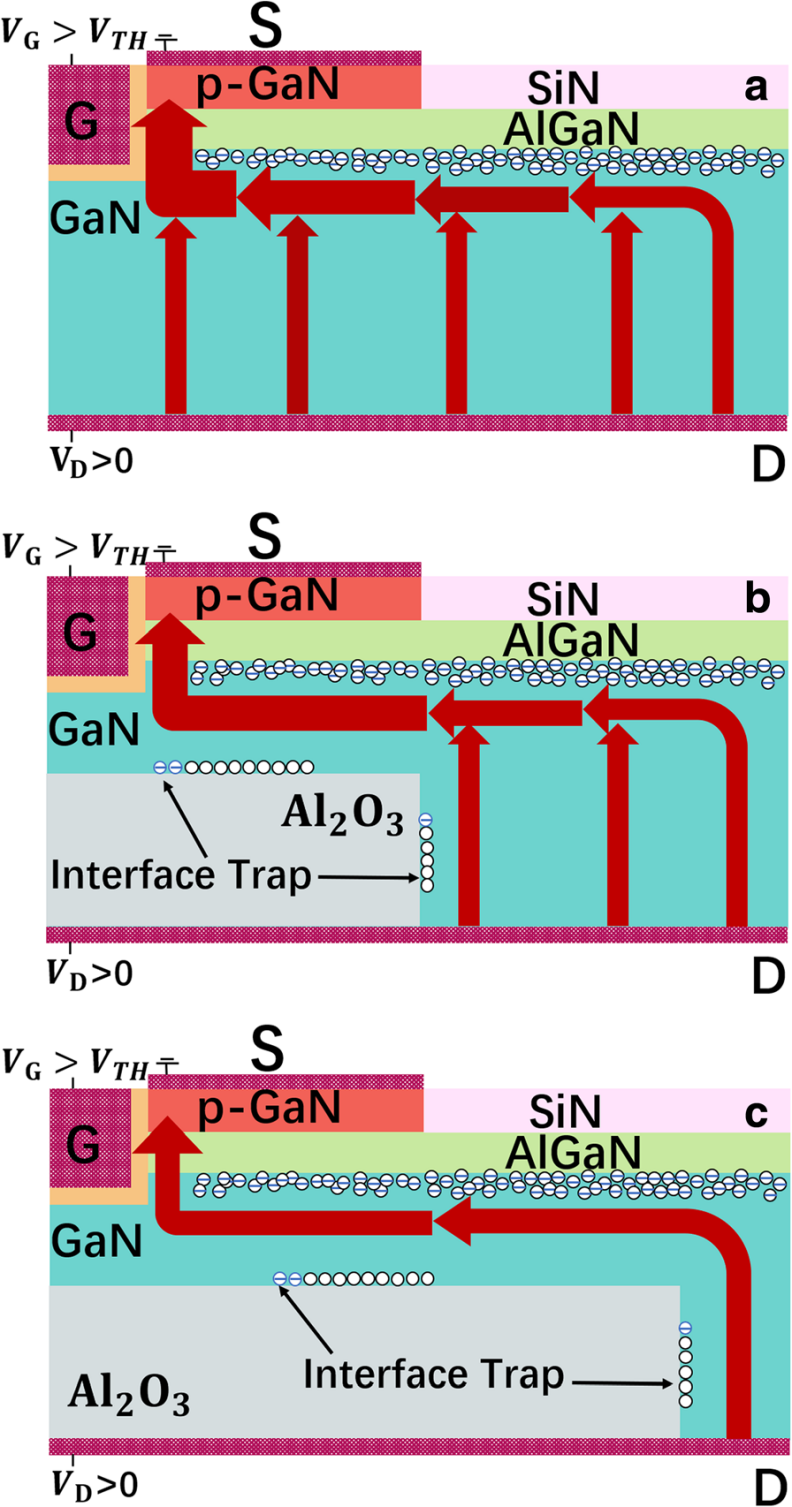
The schematic illustration of the forward current flow in **a** the device without the SP, **b** the proposed SP-VFET with a short SP, and **c** a long SP

In on-state, compared with the device without the 2-DEG channel and the SP, the proposed vertical field effect transistor with substrate pattern (SP-VFET) features a highly conductive path owing to the 2-DEG and a narrower vertical current channel that shrinks the conductance as shown in Fig. 2. In detail, thanks to the high-density 2DEG concentrated at the AlGaN/GaN interface, the lateral path of the current flow could be sustained, which partially compensates the whole device conductance. In contrast, the current transportation capability of the SP-VFET device without 2DEG channel would be influenced dramatically.

**Fig. 3:**
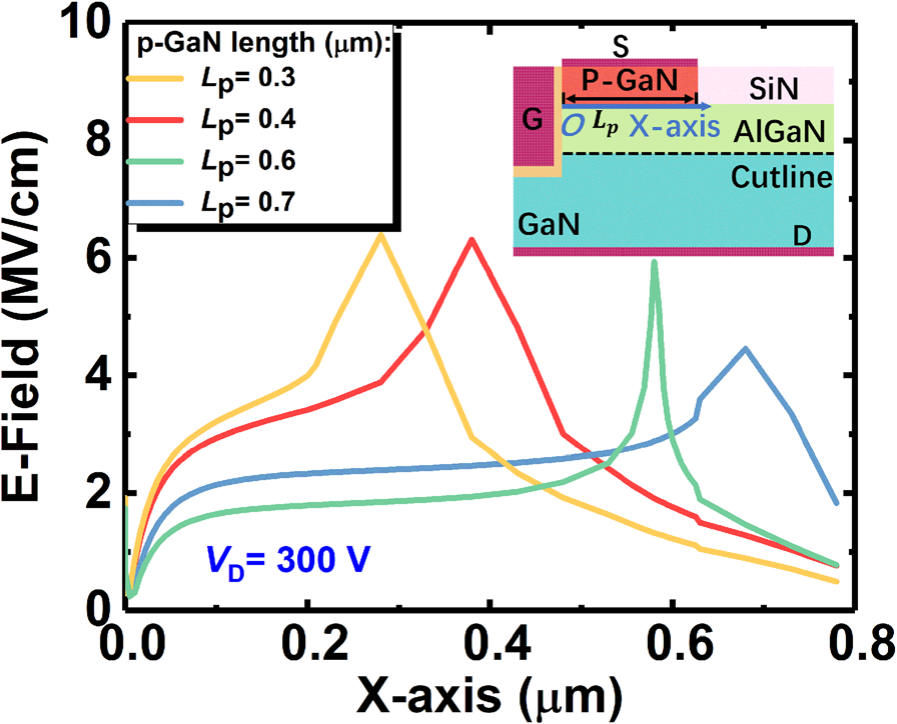
The electric field distribution of the device without the SP along the AlGaN/GaN heterojunction in different p-GaN length

The length of the p-GaN cap would not dramatically influence the concentration of electric field (E-field) until the length is longer than 700 nm by which the p-GaN almost covers the whole device surface. As shown in Fig. 3, the E-field distribution along the AlGaN/GaN interface owns a peak around the right corner of the p-GaN. The position of the peak shifts along with the varying p-GaN length, and however, keeps the same magnitude. Tiny difference of the peak value could be seen in Fig. 3 when the p-GaN cap is longer than 600 nm, because the long p-GaN cap flattens the whole E-field in the device and hereby expands the resistance of the device due to the depletion of the 2DEG.

**Fig. 4:**
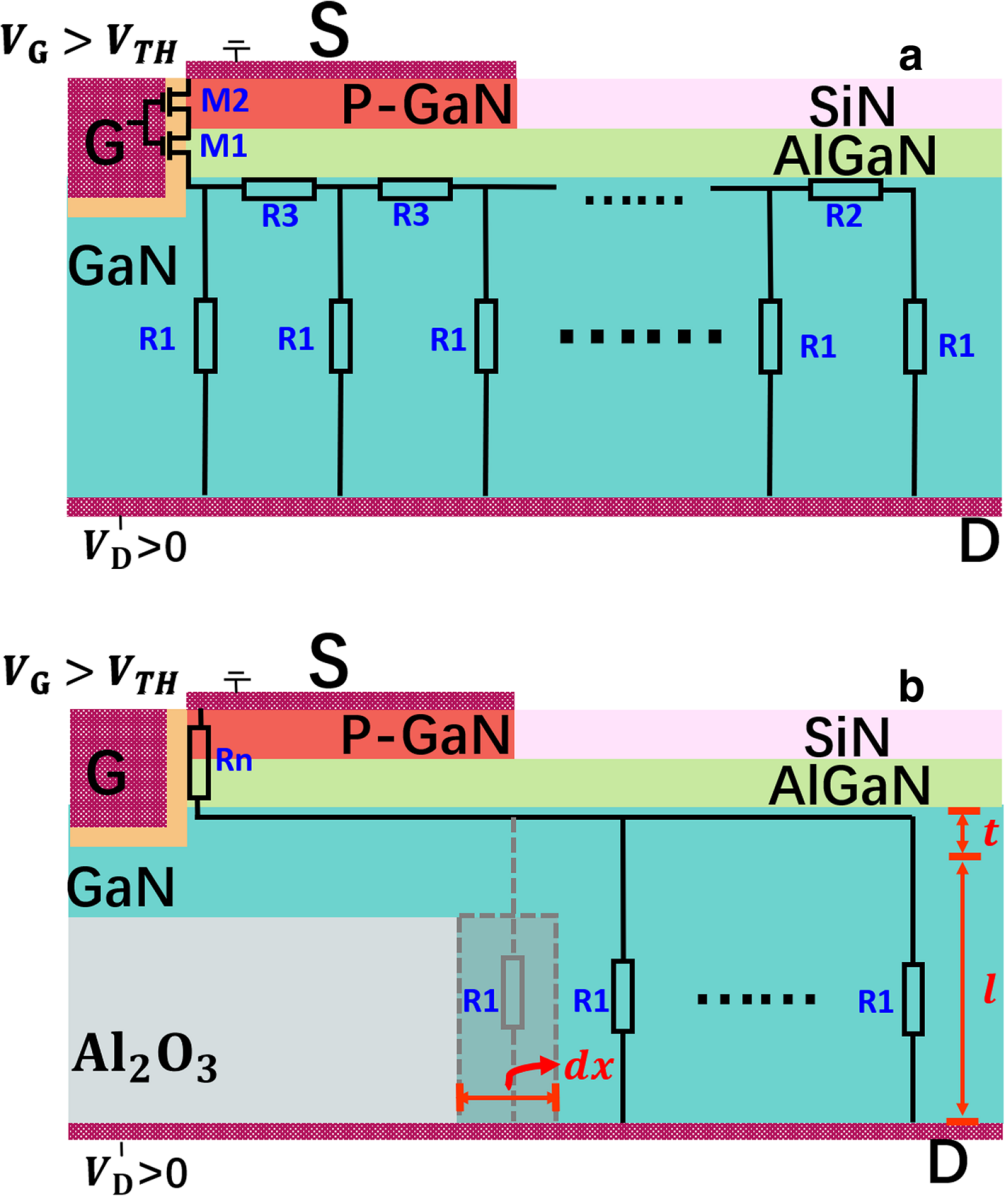
The schematic of **a** proposed on-state model without SP **b** on-state resistance network model with SP

To illustrate the influence of the simultaneously introduced p-GaN, 2-DEG, and the SP, an on-state conduction model can be built, as schematically shown in Fig. 4a. *M*_1_ and *M*_2_ are the MIS-like transistors with the conduct-channel formed in p-GaN and AlGaN respectively. *R*_1_ represents the infinitesimal part of vertical resistance in bulk GaN. *R*_2_ and *R*_3_ represent the infinitesimal resistance parts of 2-DEG channel with and without being partly depleted respectively. According to the law of resistance, *R*_1_, *R*_2_, and *R*_3_ can be obtained as

$$ {R}_1=\frac{1}{n_1 q\mu}\bullet \frac{l}{dx\bullet {W}_D} $$

$$ {R}_2=\frac{1}{n_2 q\mu}\bullet \frac{dx}{t\bullet {W}_D} $$

$$ {R}_3=\frac{1}{n_3 q\mu}\bullet \frac{dx}{t\bullet {W}_D} $$

where *n*_1_, *n*_2_, and *n*_3_ represent the electric concentration in GaN, undepleted 2-DEG, and depleted 2-DEG respectively; *q* is the electron charge and *μ* is the mobility of electron in GaN; *l* is the length of vertical conductive path and *dx* is the infinitesimal length in horizon; *W*_*D*_ is the width of the device; and *t* is the thickness of the 2-DEG. For convenience, *t* is set to be 10 nm [7]. The concentration of the depleted 2-DEG under p-GaN *n*_3_ equals the undepleted concentration *n*_1_ minus the total negative charge in the depleted p-GaN [31], which reads

$$ {n}_3={n}_2-{n}_A{x}_D $$

The p-GaN cap can be regarded as fully depleted, thus *x*_*D*_ equals 105 nm, the thickness of p-GaN. Compared with *R*_1_, *R*_2_ and *R*_3_ are much lower than *R*_1_, because of their higher electron concentration and shorter conductive path. Therefore, the resistance in the 2-DEG channel can be ignored. In addition, when the drain voltage is small and the MIS-like transistors *M*_1_ and *M*_2_ work in the unsaturated model, the on-state resistance of *M*_1_ and *M*_2_ can be regarded as an ignorable constant resistance *R*_*n*_. To simplify the calculation, the analytical form of vertical current path conductance *G*_*v*_ of vertical current path can be obtained as

$$ {G}_v={\int}_0^{L_D}\frac{1}{R_1}={\int}_0^{L_D}{n}_1 q\mu \bullet \frac{dx\bullet {W}_D}{l} $$

where *L*_D_ is the length of the device.

Therefore, the on-state resistance *R*_*on*_ can be obtained, which reads

$$ {R}_{on}=\frac{1}{G_v}+{R}_n=\frac{1}{n_1 q\mu}\bullet \frac{l}{L_D\bullet {W}_D}+{R}_n $$

When SP exists, as shown in Fig. 4b, the vertical conductive path has been blocked partially. Thus, the conductance of vertical current path can be expressed as

$$ {G}_v={\int}_{L_W}^{L_{\mathrm{D}}}\frac{1}{R_1}={\int}_{L_W}^{L_D}{n}_1 q\mu \bullet \frac{dx\bullet {W}_D}{l} $$

where *L*_*W*_ is the length of the SP.

Therefore, the corresponding *R*_*on*_ can be expressed as

$$ {R}_{on}=\frac{1}{G_v}+{R}_n=\frac{1}{n_1 q\mu}\bullet \frac{l}{\left({L}_D-{L}_W\right)\bullet {W}_D}+{R}_n $$

**Fig. 5:**
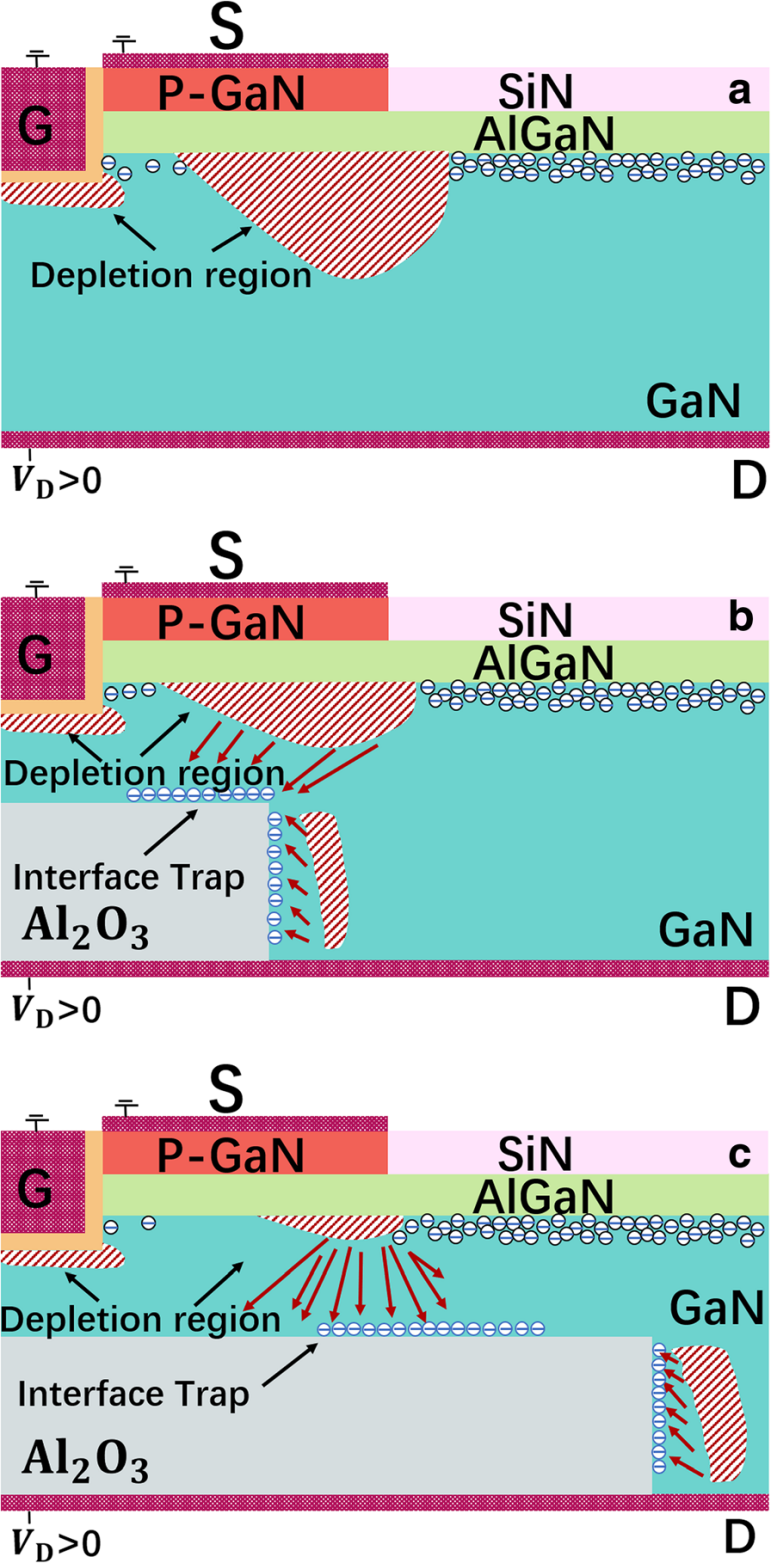
The schematic distribution of the electric field and depletion region in **a** the device without the SP, **b** the proposed SP-VFET with a short SP, and **c** the proposed SP-VFET with a long SP

In off-state, due to the capacitor-like functionality and the negatively charged interface trap, the SP would redistribute the electric field under the p-GaN cap effectively, turning out a field concentration around the SP that owns wider band gap as shown in Fig. 5. Such E-field redistribution shrinks the depletion region that appears around p-GaN and the gate, and thereby relieves the high field concentration around the p-GaN cap and the gate, which would enhance the BV of the SP-VFET remarkably. On the other hand, as mentioned above, the SP would influence the device conductance such that the negative charge introduced by the SP leads to the increase of potential energy near the gate, which accounts for the decrease of 2-DEG near the gate. As a result, a fluctuant BFOM would be achieved with varying the length and the height of the SP.

**Fig. 6:**
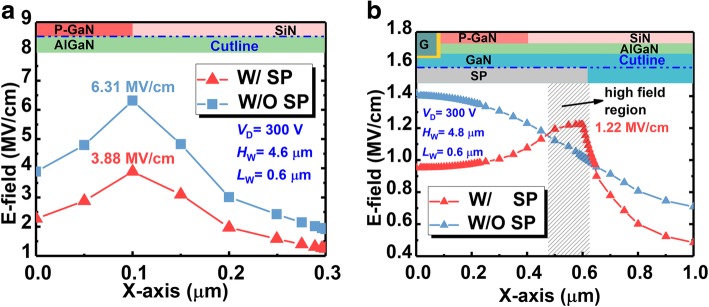
The electric field distribution of the proposed SP-VFET along the interface of **a** p-GaN/AlGaN and **b** GaN/SP

In other words, the SP could reduce the peak of the E-field around the p-GaN corner and simultaneously, attract the E-field concentrating across the SP, as shown in Fig. 6a, b. However, thanks to the higher critical E-field of the SP, such E-field concentration would not break the device, by which the SP-VFET would exhibit much higher BV.

## Result and Discussion

**Fig. 7:**
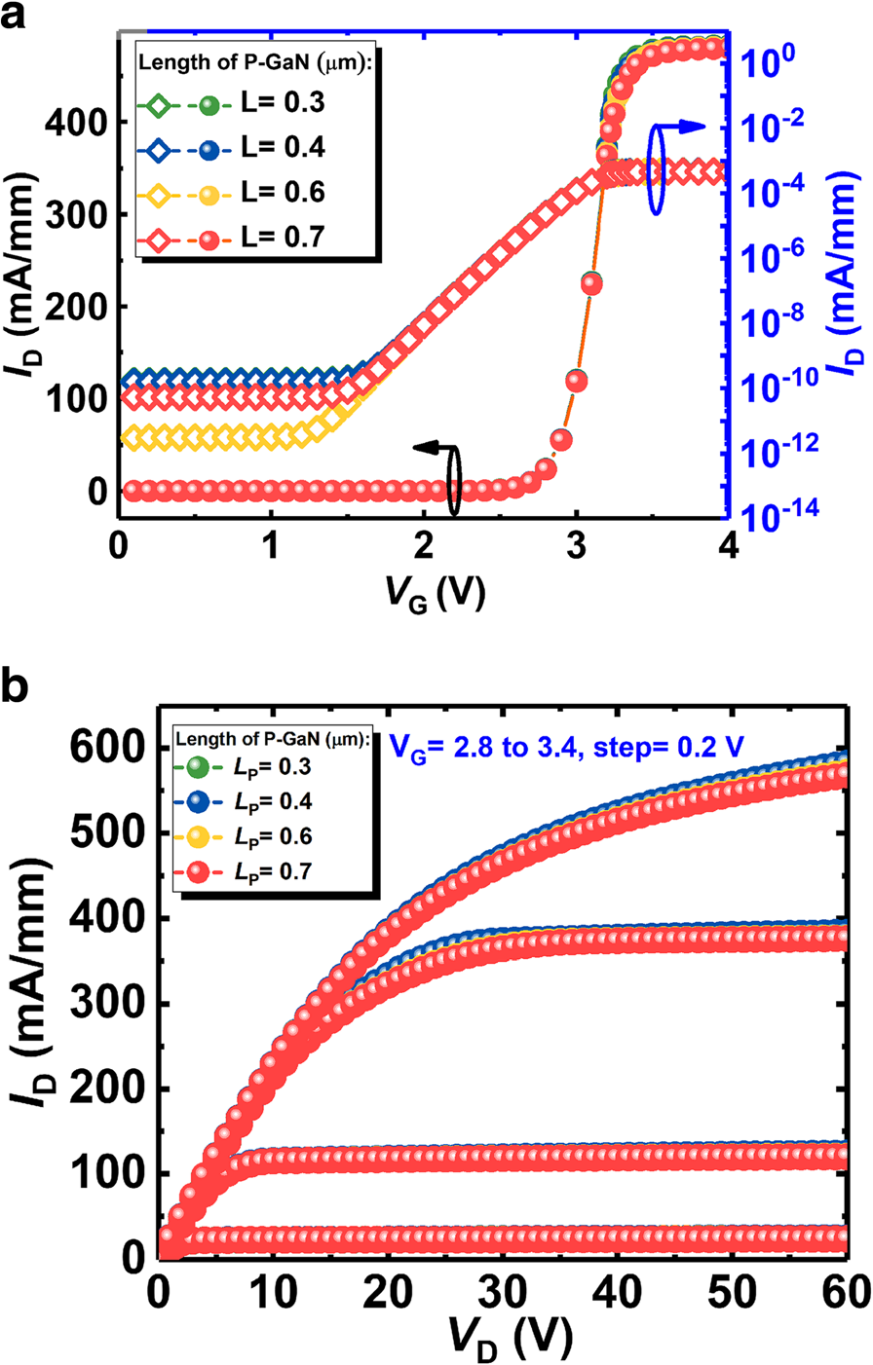
**a** The transfer curves and **b** output curves of the device without the SP in different length of the p-GaN cap

Figure 7a, b respectively shows the transfer and output curves of the proposed device without the SP. With different length of the p-GaN cap, these curves overlay each other in both subthreshold and turn-on regimes, suggesting the length of the p-GaN cap does not influence the conductance of the device without the SP. In other words, although the p-GaN cap would partially deplete the 2-DEG and hereby affect the resistance of the 2-DEG channel, the remained 2-DEG still owns a large concentration *n*_3_ that approximates the undepleted concentration *n*_2_, which is realized by optimizing the p-type concentration in p-GaN cap. Furthermore, as analyzed before, the resistance of the 2-DEG channel is rather small compared with the resistance of the n-GaN in vertical path. Therefore, the transfer curves overlay each other in Fig. 7a, b. However, in order to protect the gate from the highly concentrated E-field, such crowding should not be adjacent to the gate, which means the length of the p-GaN could not be too short. Thus, the minimum length of the p-GaN in our work is 400 nm unless otherwise stated.

**Fig. 8:**
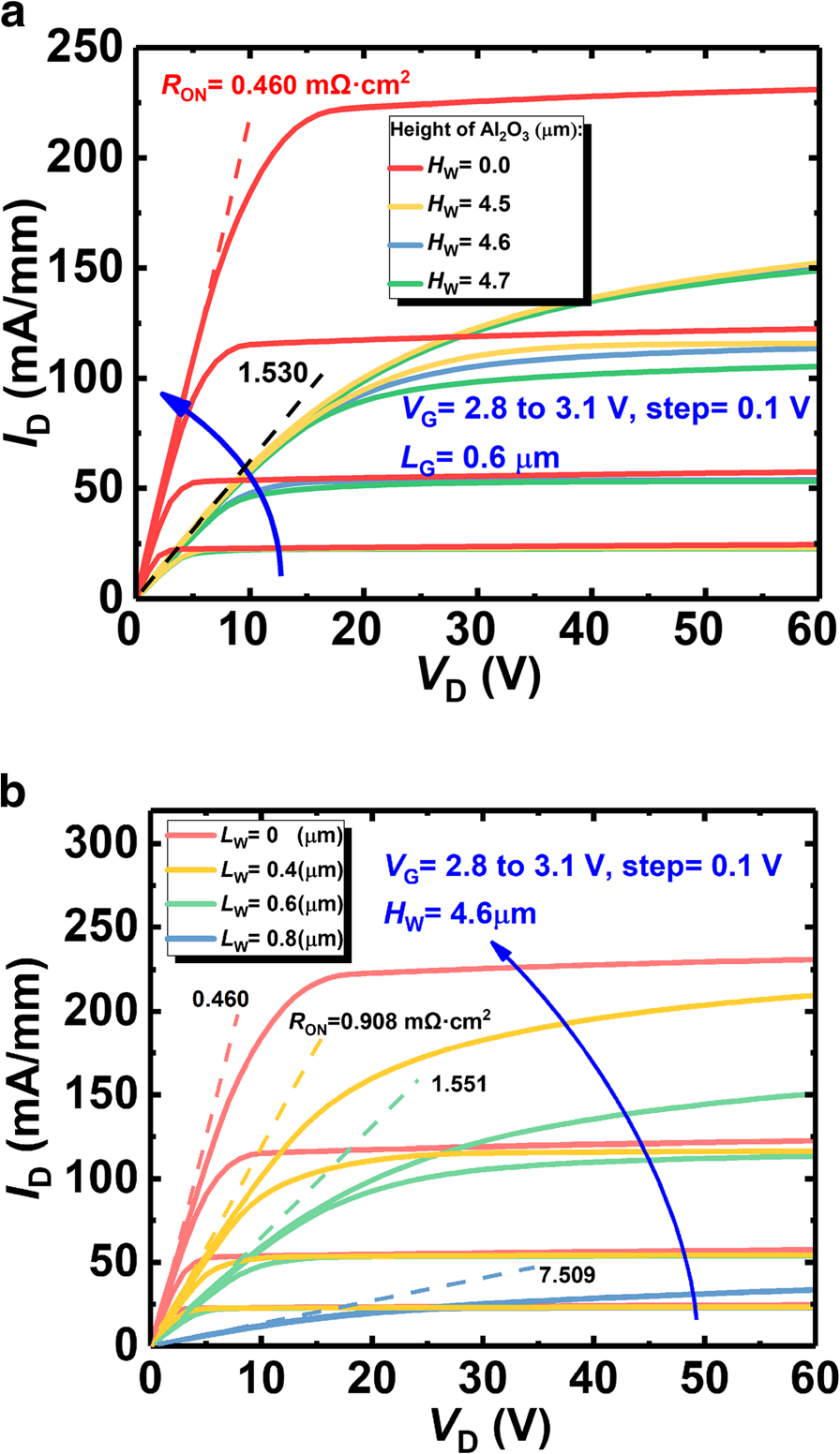
The output curves of the proposed SP-VFET with varying **a** SP width and **b** SP length compared with the device without SP

Figure 8 shows the output characteristics of the proposed SP-VFET and the VFET without the SP. It can be seen that the SP does impose the device resistance by narrowing the vertical conduction channel. In detail, the on-state resistance is independent to the height of the SP when the height is below 4.7 μm, while, significantly depends on the length of the SP which matches the mechanism that narrow vertical current path shrinks the conductance. The former independency is because the 2DEG is the main lateral conduction channel that would not be weakened by the SP within its moderate height. However, if the SP is adjacent to 2-DEG channel, the introduced negative charge around the SP will level up the energy band, resulting in the dramatical decrease of the 2-DEG concentration. Consequently, the resistance of the 2-DEG increases and the total on-state resistance *R*_*on*_ increases accordingly. Besides, the later dependency comes from the remarkably boosted resistance in the vertical channel as aforementioned. Furthermore, it should be noted that the lattice defects in 2-DEG induced by high SP limit the height of SP.

**Fig. 9:**
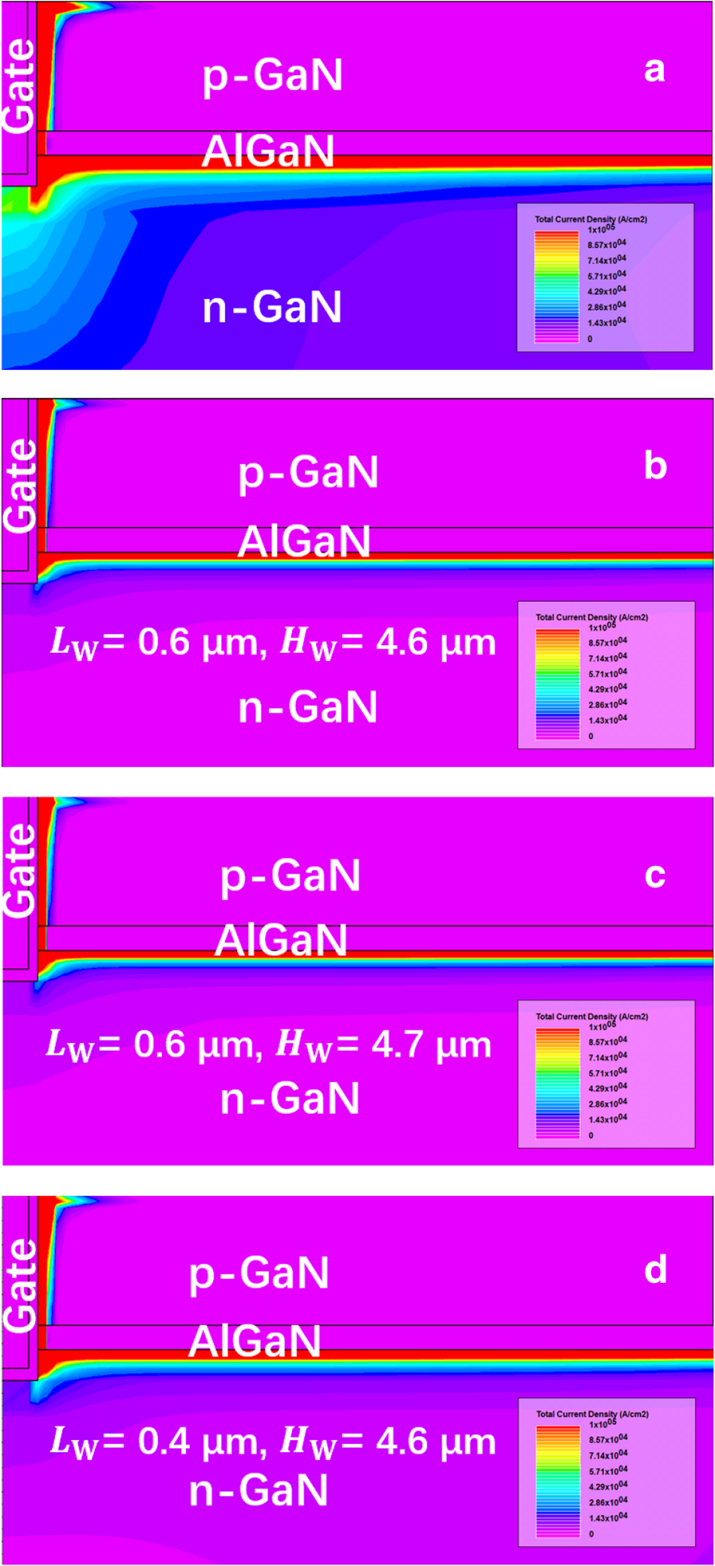
The current density of **a** the device without the SP and **b**–**d** the proposed SP-VFET with different SP geometric parameters

Figure 9 shows the details of the current density distribution around the gate of the VFET devices with or without the SP, wherein the VFET without the SP has a higher current transportation capability which keeps in line with Fig. 8a. And oppositely, the SP-VFET shrinks the current conduction by narrowing the vertical channel. Meanwhile, the detailed figures clearly illustrate that the current in the lateral channel is transported by the 2DEG, and the total current density changes slightly with growing SP height, which is also demonstrated in the mechanism section. The results indicate the lateral channel resistance is not imposed notably by the SP within moderate height.

**Fig. 10:**
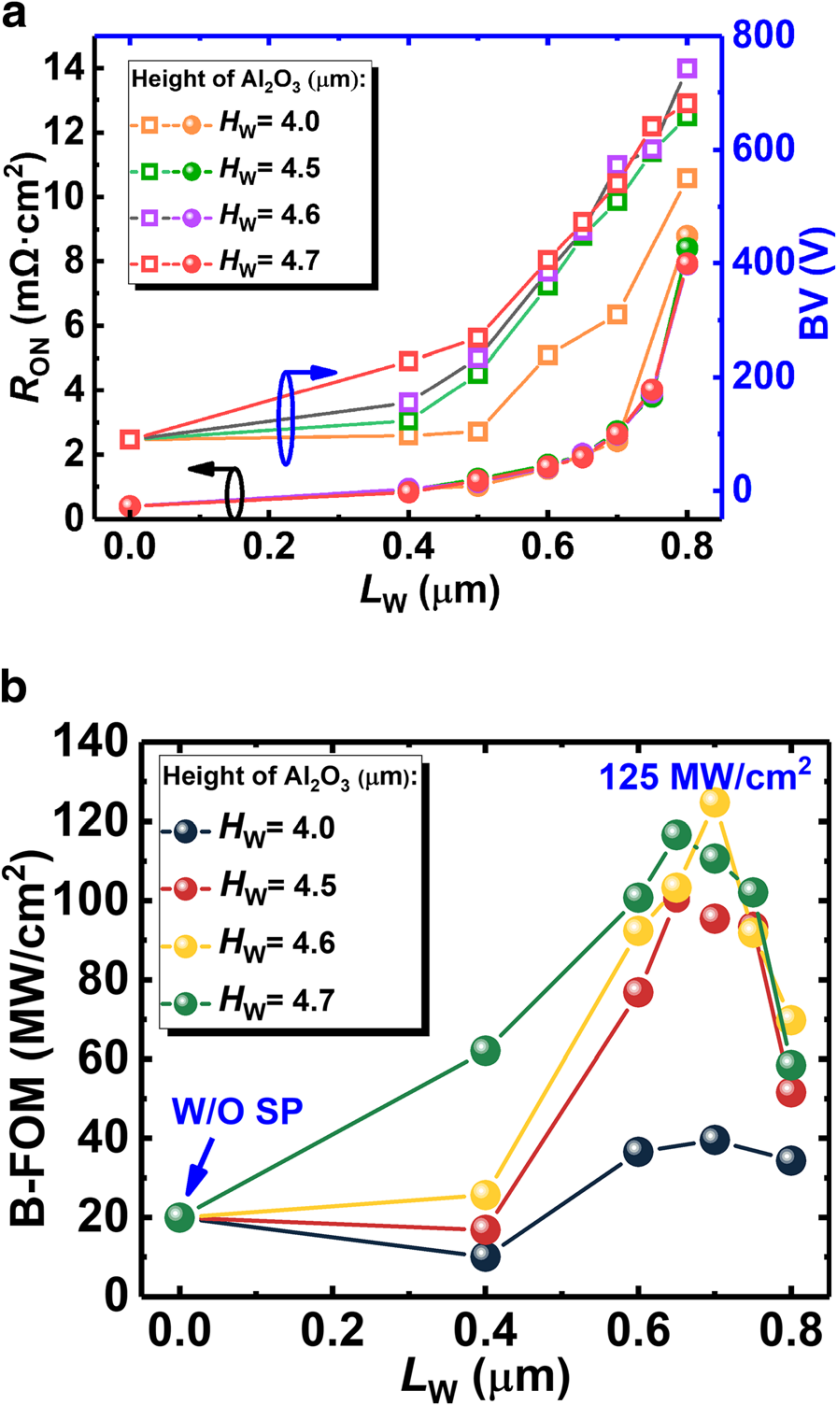
**a** The extracted on-state resistance as well as BV, and **b** the according BFOM of the proposed SP-VFET in different SP length and width

Figure 10a shows the extracted on-state resistance and the corresponding BV. The resistance of the SP-VFET increases with longer SP. And especially, the curve of the resistance versus SP length exhibits a hyperbolic trend, and the gradient of the curve increases with the longer SP length. As analyzed before, *R*_*on*_ varies with different SP length *L*_*W*_ in a form of hyperbolic function, which matches the simulation result. Moreover, the curve of the resistance with different SP heights overlays each other as the height is lower than 4.7 μm, suggesting that 2-DEG channel is the main lateral conductive path and the 2-DEG channel is not affected, as mentioned above.

Figure 10b shows the calculated BFOM of the SP-VFET. Owing to the different increasing rate of BV and on-state resistance, the BFOM of all the devices firstly grow and then drop after the length of the SP longer than 400 nm. A peak BFOM of 125 MW/cm^2^ is achieved when the length reaches 700 nm and the height reaches 4.6 μm. Compared with the device without the SP, the proposed SP-VFET performs more than six times better in terms of the BFOM.

This improvement is achieved owning to the suppression of the high E-field under the p-GaN, thanks to the negatively charged interface trap around the SP. The interaction, which occurs between the trapped negative charge on the interface of the SP and the depletion region around the p-GaN, forms a new distribution of E-field mainly towards the trapped charge. According to the Gauss’ law, the electric flux is limited by the charge encircled. Thus, the introduced E-field will affect the electric flux toward elsewhere. As the negative charge of depletion region is the main source for the crowed E-field around p-GaN, the E-field introduced by the trapped charge will play a role in suppressing the E-field crowed around p-GaN, and consequently, BV is enhanced. Specifically, when the SP length is lower than 400 nm, the negative charge introduced by SP is far away from the depletion region. Thus, the E-field formed between the depletion region and trapped negative charge is too small to play a role in affecting the crowed E-field under p-GaN. And as a result, the BV of the device grows slightly. However, as the SP length is higher than 400 nm, owing to the more trapped negative charge on the interface of the SP and shorter distance between the depletion region and trapped negative charge, the E-field between the depletion region and trapped negative charge is enhanced, leading to the growth of the BV.

**Fig. 11:**
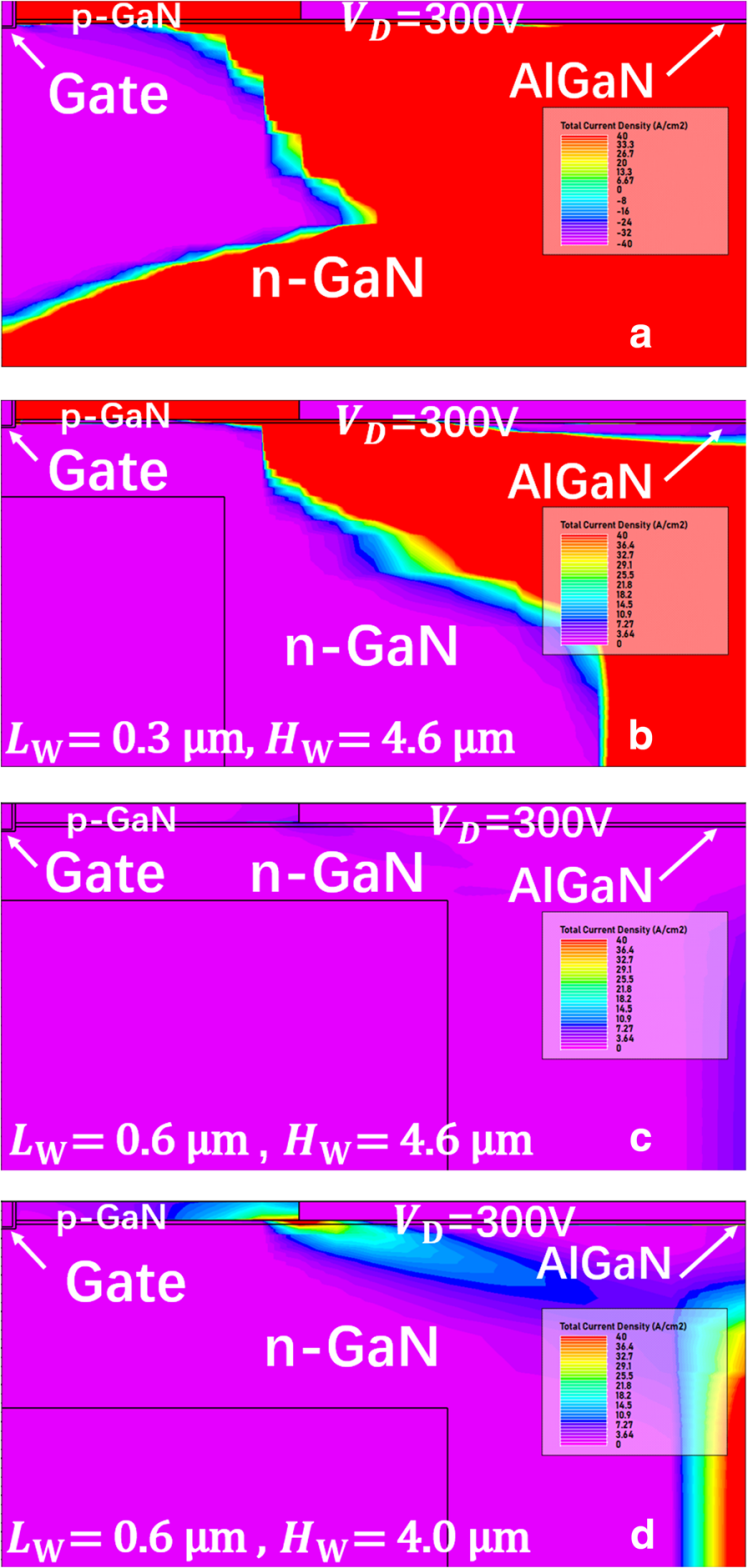
The leakage current density of **a** the device without the SP and **b**–**d** the proposed SP-VFET with different SP geometric parameters

Additionally, the region around the SP is tremendously depleted due to the negative charge introduced by SP. And as shown in Fig. 11, with the longer SP, the vertical leakage current path constricts in width owing to the depleted region squeezing to the device edge, which also block the leakage current, and consequently enhance the BV. Therefore, the BV rises remarkably with the increasing SP length.

**Fig. 12:**
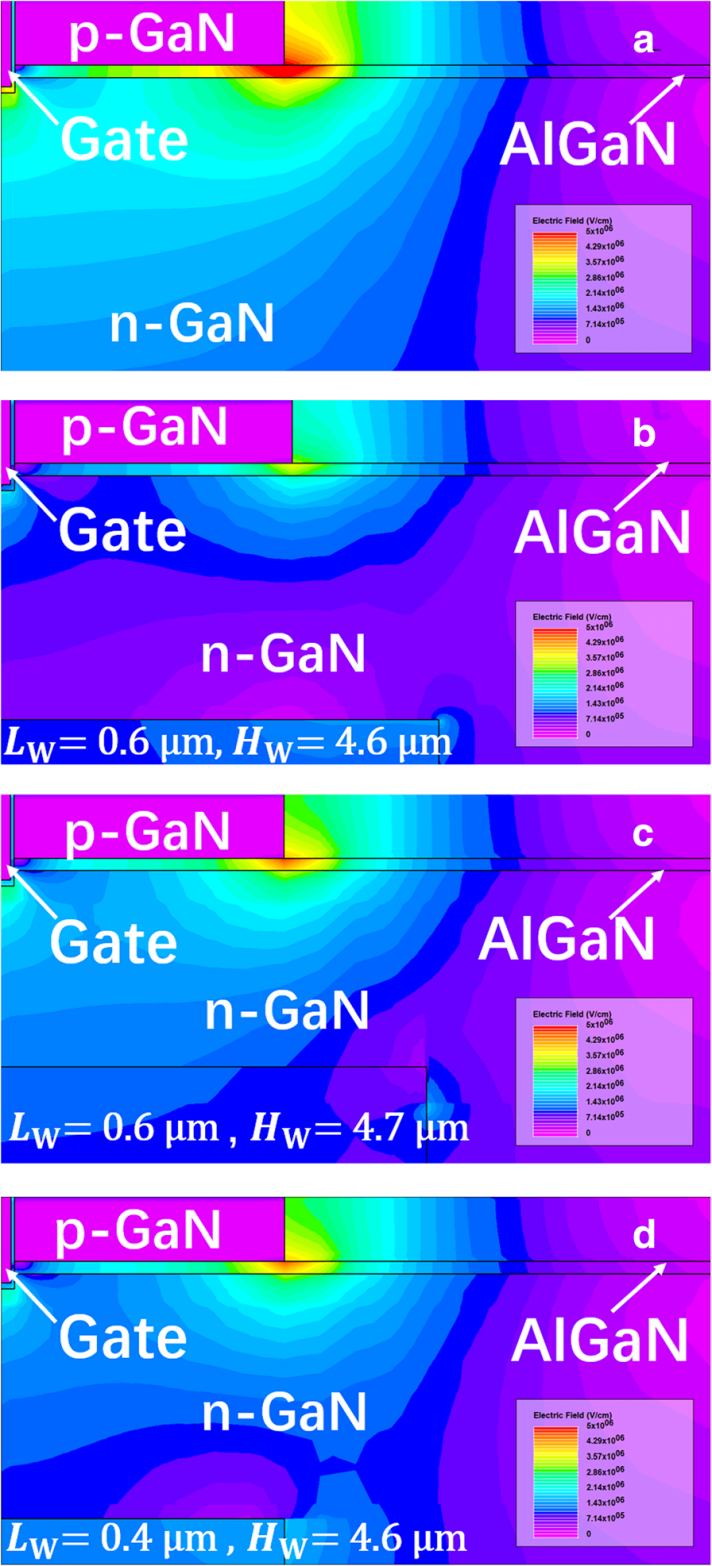
The E-field distribution around the gate of the VFET **a** with or **b**–**d** without the SP when the drain voltage is 300 V

Figure 12 shows the simulated E-field distribution in the SP-VFET when the drain voltage is 300 V, where apparently the SP induces other new E-field concentration points, meaning that the peak E-field region under the p-GaN is suppressed. Compared with the E-field distribution in the devices with different SP height, the increase of SP length suppresses the congregation of E-field and consequently enhances the BV more efficiently.

**Fig. 13:**
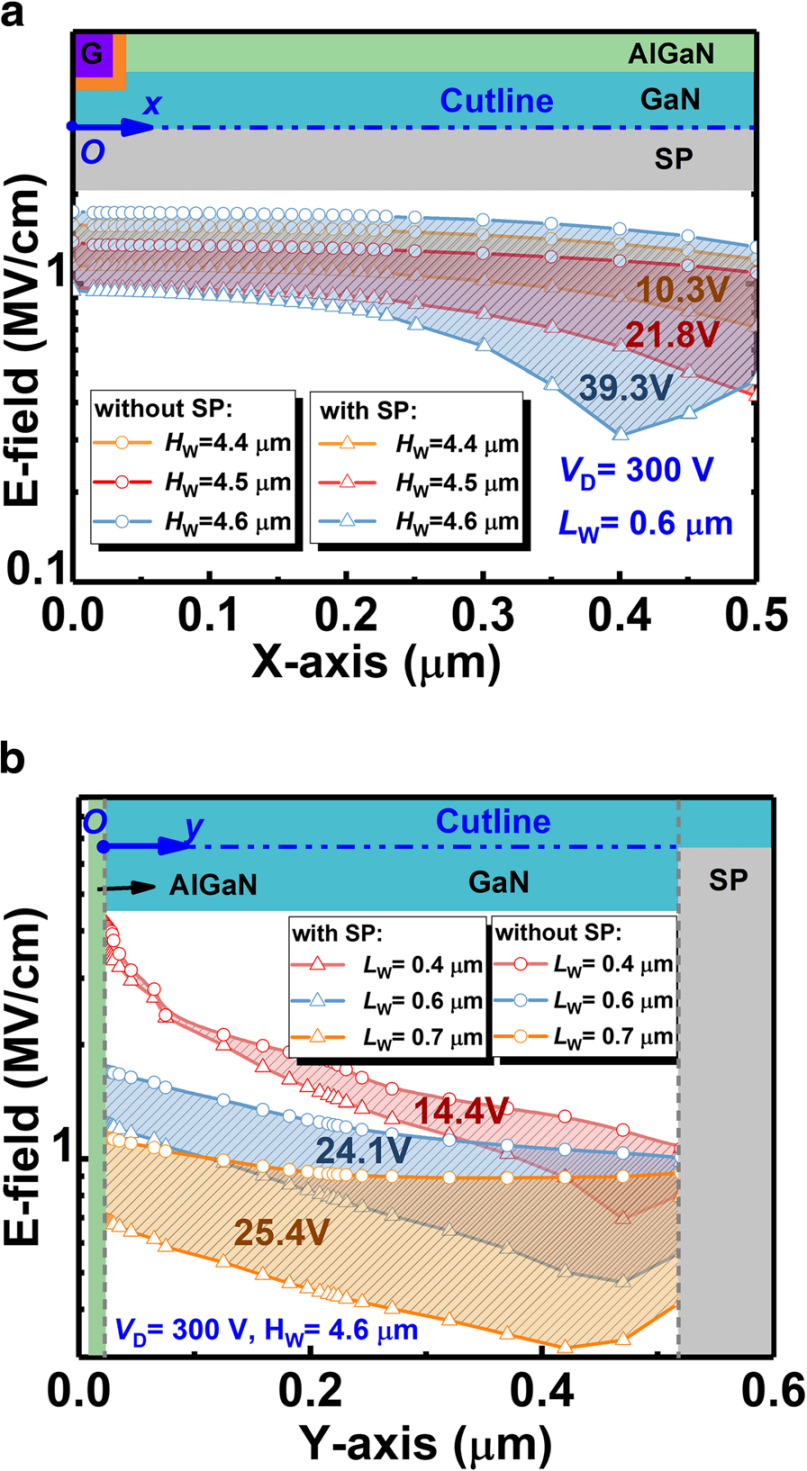
The E-field distribution **a** horizontally and **b** perpendicularly extracted along the cutline of the SP edge in the SP-VFET

Such flattened E-field could also be observed explicitly in Fig. 13, in which the E-field distributions along the horizontal and perpendicular edge of the SP (see the cutline) are plotted. As demonstrated in polychrome Fig. 12, it can be seen in Fig. 13 that higher and longer SP plays a more and more effective role in reconstructing the E-field under p-GaN, and, attracting E-field to concentrate across the SP edge. This redistribution counteracts the E-field around vulnerable p-GaN. Thus, the BV of the device is enhanced, boosting the B-FOM of proposed SP-VFET.

## Conclusion

In this work, a novel enhancement-type GaN vertical FET (SP-VFET) with 2DEG channel and substrate pattern for improving the BFOM thereof is proposed and investigated. Verified by experimentally calibrated simulation implemented with ATLAS, it is the SP that relieves the E-field peak under the p-GaN, and simultaneously, attracts new E-field concentration across the SP that owns higher critical E-field. Consequently, the BV of the proposed SP-VFET is boosted with a moderately increasing on-state resistance due to the 2DEG compensation. The BFOM of the SP-VFET therefore is enhanced six times better than that of the device without the SP when the SP length and height are 700 nm and 4.6 μm respectively, rendering the promising potential of the proposed SP-VFET in high-density power integration.

## Abbreviations

*n*_A_: Doping concentration of p-type GaN
*n*_D_: Doping concentration of n-type GaN
2DEG: Two-dimensional electron gas
Al_0.23_GaN: Aluminum gallium nitride with a mole fraction of 0.23 for aluminum
Al_2_O_3_: Aluminum oxide
BFOM: Baliga’s Figure-Of-Merits
*D*_*SP*_: Interface trap density of the substrate pattern
E-field: Electric field
*E*_*T*_: The difference between the conduction band and interface-trap energy-level
FET: Field effect transistor
GaN: Gallium nitride
HEMT: High electron mobility transistor
*H*_G_: Height of the gate
*H*_W_: Height of substrate pattern
*L*_*D*_: Length of the device
*L*_G_: Length of the Gate
*L*_*P*_: Length of the p-GaN cap
*L*_*W*_: Length of the substrate pattern
SiN: Silicon nitride
SP: Substrate pattern
SP-VFET: Vertical field effect transistor with substrate pattern
*W*_*D*_: Depth of the device
*σ*_p_: Polarization charge
